# Re-rupture rate and the post-surgical meniscal injury after anterior cruciate ligament reconstruction with the *Press-Fit-Hybrid®-*technique in comparison to the interference screw technique: a retrospective analysis of 200 patients with at least 3 years follow-up

**DOI:** 10.1007/s00402-022-04368-7

**Published:** 2022-03-15

**Authors:** Richard Volz, Gudrun H. Borchert

**Affiliations:** 1 Joint Practice Renz‚ Volz and Loewe, Center for Specialized Joint Surgery, Haegerstraße 4, 88662 Ueberlingen, Germany; 2 Dr. Borchert Medical Information Management, Egelsbacher Str. 39e, 63225 Langen, Germany

**Keywords:** ACL, *Press-Fit-Hybrid®-technique*, Interference screw, Meniscal injury, Knee stability, Hamstrings autograft

## Abstract

**Background:**

There is currently no consensus regarding the preferred surgical procedure for the reconstruction of anterior cruciate ligament (ACL). The interference screw technique is widely used, but has been associated with a risk of graft damage. The *Press-Fit-Hybrid®*-technique is one of the alternatives for biological ACL-reconstruction with minimal implant requirements. The hypothesis of this retrospective analysis is, that the *Press-Fit-Hybrid®*-technique leads to better results with respect to re-rupture rate and secondary meniscal lesion than the interference-screw-technique.

**Methods:**

To compare the re-rupture rate of the interference-screw-technique (IF) used until 2015 with the currently used *Press-Fit-Hybrid®*-technique (PFH), the last 100 patients of the IF-group and the first 100 patients of the PFH-group were retrospectively analyzed. Primary outcomes were re-rupture rate, complications and secondary meniscal injury. Additionally, laxity, Lachman and Pivot-shift and range of motion were evaluated.

**Results:**

A mean follow-up of 4.2 and 5.3 years revealed 4% and 9% re-rupture rates and 1% and 2% complication rates in the PFH- and IF-group, respectively. In the PFH-group there were no re-ruptures in patients older than 23 years. Secondary meniscal injury post-surgery was 6% and 9% for the PFH and IF-group, respectively. Knee stability was similar in both groups. Range of motion was significantly better in the PFH-group, with 136° of flexion, 6 months after surgery.

**Conclusion:**

For ACL-reconstruction the *Press-Fit-Hybrid®-technique* is an alternative new method. Low level of secondary meniscal lesions after surgery and high stability, is known to prevent later arthrosis of the knee. The encouraging observed trend of the reduction of the re-rupture rate in revision surgery and in young patients using the *Press-Fit-Hybrid®-*technique in comparison to the interference-screw-technique must be confirmed with further studies**.**

**Level of evidence:**

Therapeutic Level III**,** retrospective cohort study.

**Supplementary Information:**

The online version contains supplementary material available at 10.1007/s00402-022-04368-7.

## Introduction

The aim of anterior cruciate ligament (ACL) reconstruction is to reduce the onset of secondary degenerative changes and to restore the ability to perform sport activities on a level comparable to that before incurring the injury [6]. Appropriate indications and precise surgical techniques are crucial to ensure postoperative stability [18]. The restoration of normal knee joint kinematics reduces the risk of subsequent meniscal lesion and improves healing after meniscal refixation [27, 45]. The most commonly used method is the interference-screw-technique, but it has been associated with risks of intra-operative graft damage and secondary graft damage caused by screw misplacement [41]. A mainly hardware free—more biological method was surged for, which additionally avoids known problems in the case of revision [41]. Press-Fit-Fixation is one of the existing alternatives [3, 4, 6, 15–17, 30, 34, 36, 41], and is a term used to describe a set of alternative procedures [3, 4, 6, 15–17, 30, 34, 36, 41]. In 2003 Pässler et al. [34] reported that in addition to Bone-Patella-Tendon-Bone (BTB) and quadriceps tendons, semitendinosus tendons could also be used for the press-fit-technique. They observed, that the use of the semitendinosus tendon reduced both, donor site morbidity and the rate of arthrosis, while leading to similar re-rupture rates [36]. A tibial tunnel enlargement was reported due to the fact that no impaction of the tunnels was carried out [36]. Galla et al. described 2004 a press-fit version, using hamstring tendon, where a bone block was used on the femoral side additional to a cross pin and screws on the tibial side [17].

Felmet [15] described 2010 an ‚All-Press–Fit’-ACL-reconstruction with semitendinosus/gracilis tendons. 46 patients were treated and very good Lachman-Scores and Pivot-Shift-Test-results were reported. There was only 1 re-rupture in the first 7 months [15]. This technique was thoroughly revised from 2010 to 2012 by *BIOMEDIX®* in co-operation with Dr. Missalla (Ortho-Klinik Rhein-Main) to create the next generation *Press-Fit-Hybrid®*-technique. The new, standardized *Press-Fit-Hybrid®*-technique is characterized by (a) the use of an atraumatic AlphaLock®-Turbo-Cutter to simultaneously generate a tunnel and a cylinder, (b) femoral/tibial fixed stops (avoiding tibia fractures) for a safe workflow, (c) calibrated impaction of the femoral/tibial implant sites to achieve ideal Press-Fit conditions and (d) exact tunnel dimensions for optimal primary stability. The bone cylinder is impacted close to the joint to maximize the contact between the bone cylinder and vital bone, whilst avoiding graft damage. The differences between the *Press-Fit-Hybrid®/Volz-*technique and the *Press-Fit-Hybrid®*/*Missalla*-technique are modified graft preparations and distal fixation. The tibial fixation includes not only a press-fit fixation of the bone cylinder, but also an additional bollard screw to which the high tensile threads are attached.

Parallel surgeries with both techniques showed promising results for the *Press-Fit-Hybrid®*-technique, thus the surgery technique was changed after a trial period.

The hypothesis of this retrospective analysis with 200 patients and at least 3 years follow-up is, that the *Press-Fit-Hybrid®*-technique produces better results with respect to re-rupture rates and secondary meniscal lesions than the interference-screw-technique used up to 2015. To our knowledge, the comparison of these techniques for ACL-reconstruction has not been previously described.

## Methods

### Patient data

Ethical approval for the study was obtained from the “Landesärztekammer Baden-Württemberg” (F-2019-100). An informed consent was signed by the patients included.

*Inclusion criteria were* at least 3 years follow-up and 18 years of age, not more than 1 reconstruction of the ACL on the knee under investigation before this surgery and use of semitendinosus/gracilis tendon for reconstruction. Meniscal suturing, partial meniscectomy and cartilage smoothing were considered permissible.

*Exclusion criteria were* 2 or more reconstructions of the ACL on the knee under investigation, collateral ligament reconstruction, use of semitendinosus tendon alone or quadriceps tendon for reconstruction.

The last 100 operated patients from the IF-group and the first 100 patients from the PFH-group, were included.

### Surgical procedure

All surgical procedures were carried out by the first author at the Stockach hospital, Stockach, Germany and carried out as an anatomical single bundle reconstruction with the AM-portal-technique. Hamstring tendon was used, because the donor site morbidities are lower [9] and a sling can be created for graft fixation.

#### General surgical procedure (same for both methods)

The patient is placed in a dorsal recumbent position with the leg in a holder. A pre-operative single i.v. antibiosis with 1.5 g cefuroxime is administered. The operation area is disinfected with Cutasept (GBode Chemie, Hamburg, Germany) three times and covered with sterile single-use waterproof drapes. A previously applied thigh tourniquet is closed to a pressure of 350 mmHg. A puncture incision is made over the lateral soft spot and blunt trocar is inserted into the upper recess. The joint is filled with irrigation solution. The ACL stump is resected to expose the lateral posterior edge of the notch. Temporarily concluding the arthroscopic procedure.

A 25 mm long skin incision is made over the pes anserinus followed by sharp dissection through the subcutis with meticulous hemostasis down to the Sartorius fascia. A blunt longitudinal split of the Sartorius fascia is performed. The semitendinosus and then the gracilis tendons are dislocated with an Overholt clamp. The tendons are freed from branches and stripped with an open tendon stripper.

#### Interference-Screw-technique

The two tendons are freed from muscle tissue and each is doubled or tripled to form a quadruple/six-fold tendon graft. This is securely reinforced both proximally and distally over 3 cm with a size-0-Vicryl thread. This results in a graft with approximately 11 cm length and with an approximate diameter of 8 mm. Arthroscopy is now resumed. With 130° flexion of the knee joint, the offset (e.g., 6 mm) targeting device is attached to the lateral rear notch edge at 9:30/2:30 and a drill wire (e.g., 2.3 mm) is inserted. A headspace drill (e.g., 8 mm) is used to over-drill to a depth of 30 mm. The correct position of the drill channel is checked. Then the tibial targeting device is inserted with a drill wire (e.g., 2.3 mm) so that it is centrally positioned in the area of the tibial ACL stump. A headspace drill (e.g., 7 mm) is used for over-drilling and a compacting drill (e.g., 8 mm) for compaction. The correct position of the drill channel is checked. Transtibial removal of the thread follows. Then the graft is attached by drawing it through the tibial tunnel into the femoral drill tunnel to a depth of 30 mm. It is then notched and screwed clockwise/counterclockwise with a BIOSURE interference screw (e.g., 8 × 25 mm, Smith + Nephew Inc. Andover, MA USA), over the Nitinol-guide-wire (Smith + Nephew Inc. Andover, MA USA) with good tension on the graft, ensuring a very stable fit. The knee joint is flexed 30 times between 0 and 90°. In the 20° flexion position, e.g., 9 × 30 mm MILAGRO interference screw (Mitek, San Diego; CA USA) is applied to the nitinol guide wire with good tension on the graft, ensuring a very stable fit at tibial side. An intra-articular check for optimal graft position, sufficient tension and absence of notch impingement at full extension is then conducted. Finally, a partial synovectomy in all sections is applied to reduce pain, and the joint is extensively irrigated. A 10Ch Redon drainage (Fa. B. Braun AG, Melsungen, Germany) is inserted into the upper recess and 20 ml Naropin 0.75% is applied intraarticularly and subcutaneously. The skin is sutured with the Donati single button suture technique. Over the pes anserinus, a 12Ch Redon drainage (Fa. B. Braun AG, Melsungen, Germany) is inserted with suturing of fascia, subcutis and dermis in intra-cutaneous technique. A final check for complete extension is performed and sterile, elasto-compressive wound dressings are applied.

#### *Press-Fit-Hybrid*®-technique

**Fig. 1:**
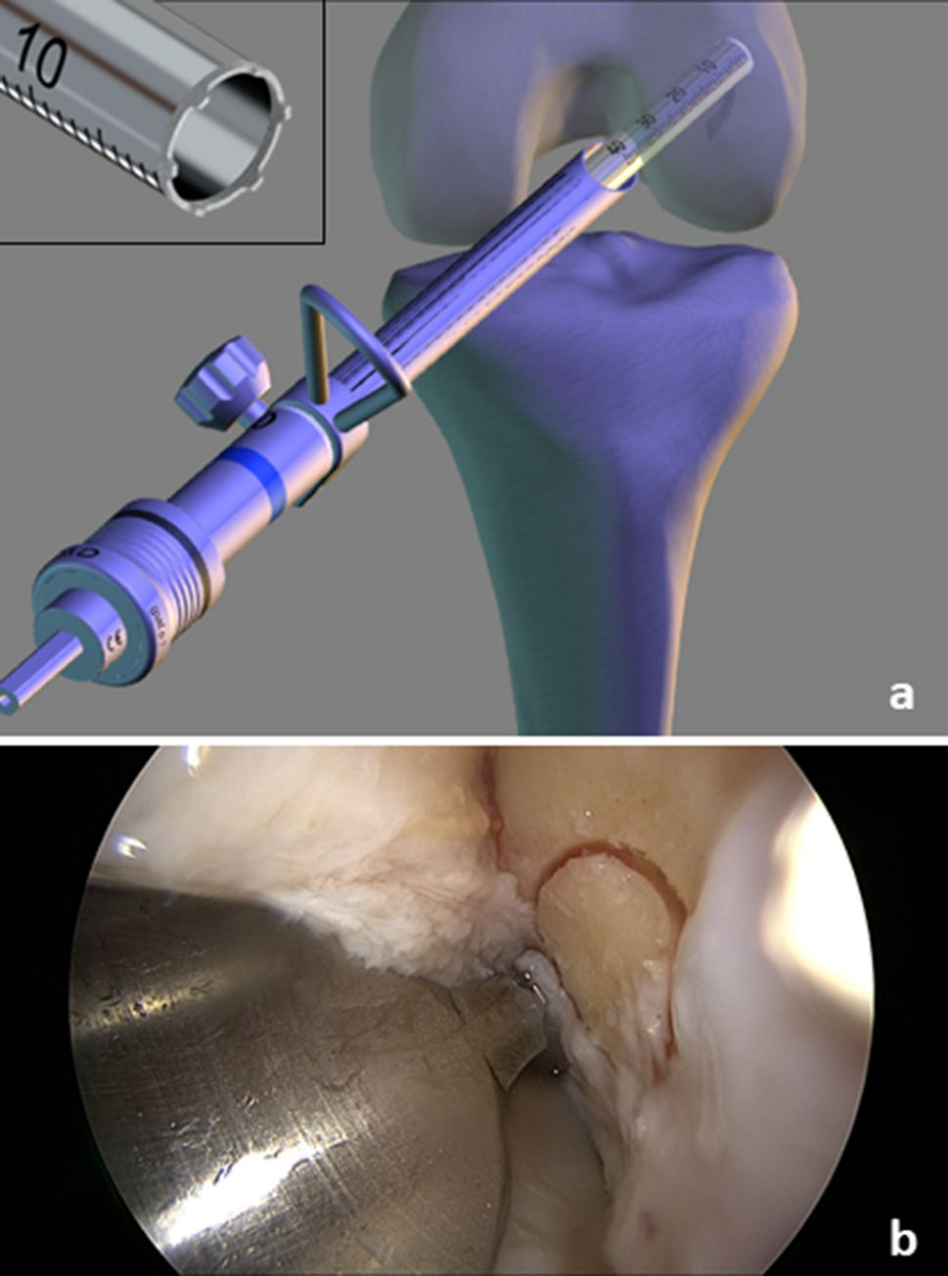
Femoral bone tunnel procedure: **a** bone tunnel target device with hallow saw in insert; **b** bone after bone tunnel drilling

**Fig. 2:**
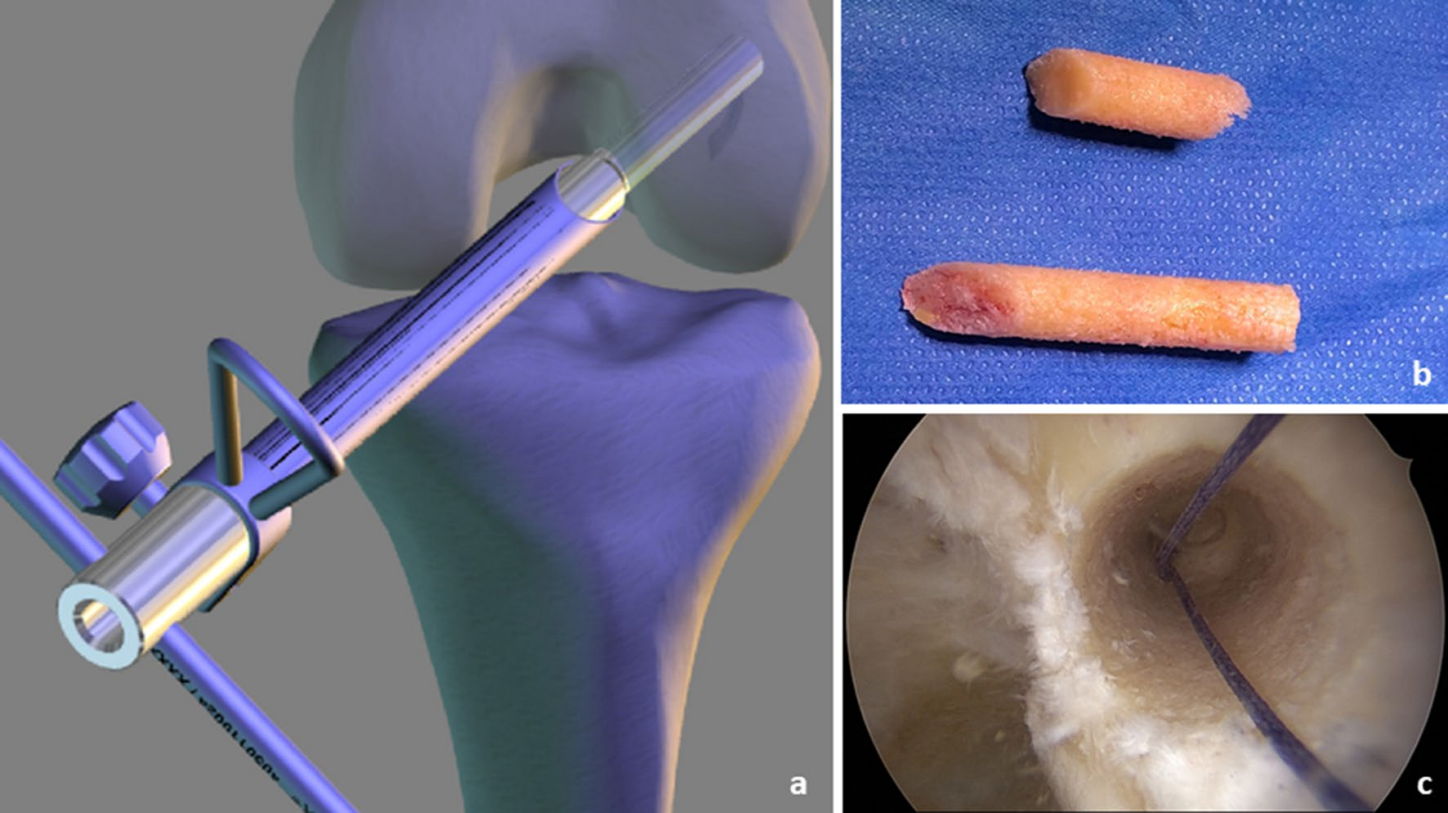
Extraction of bone block: **a** bone block extractor; **b** harvested bone blocks (upper for femoral, lower for tibial side); **c** bone tunnel after bone block harvest. The tunnel is not necrotic, due to diamond hallow saw used for this procedure

**Fig. 3:**
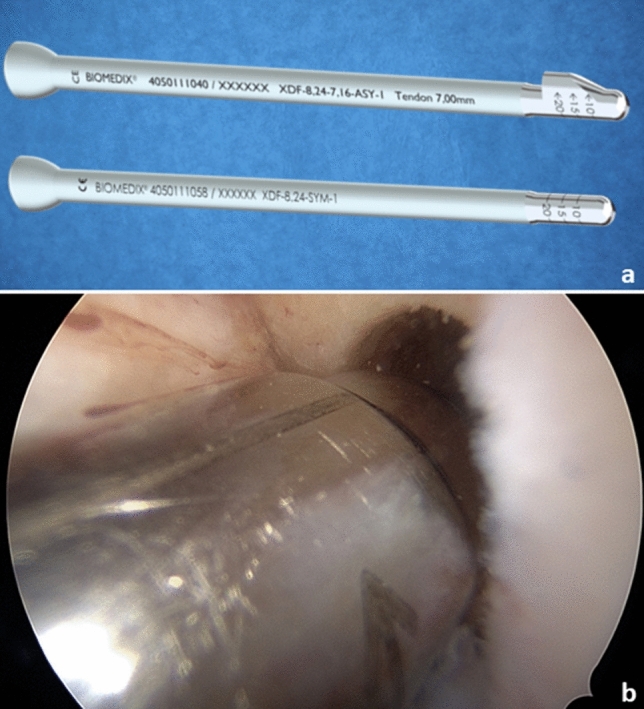
Dilatation of bone tunnel: **a** instruments for dilatation: upper: asymmetric dilatator, lower symmetric dilatator; **b** procedure of dilatation

The tendons are freed from muscle tissue and placed through the sling of an ultra-button (Smith & Nephew Inc. Andover, MA USA) to form a six-fold tendon graft. The ends are bound and reinforced with sutures—proximally 3 cm with a Vicryl size 0 thread (Ethicon, Norderstedt, Germany) and distally 4 cm with a HiFi-suture-loop (Conmed Deutschland, Groß-Gerau, Germany) and a 5-Ethibond thread (Ethicon, Norderstedt, Germany). The result is a graft that is approximately 9 cm long with a diameter of about 9 mm. The arthroscopic procedure now resumes. The bone tunnels are NOT drilled with a headspace drill, but rather with a diamond *AlphaLock®-*Turbo-Cutter (*BIOMEDIX*®, Dietzenbach, Germany, Fig. 1), that generates both the bone tunnel (Fig. 2) and the bone cylinder (Fig. 2) in a single procedure. With 130° flexion of the knee joint, the offset targeting device (Fig. 1) is attached to the lateral posterior edge of the notch at 9:30/2:30. In this manner, the tunnel can be drilled in 2–3 s thus, avoiding necrosis of the bone (Figs. 1 and 2). The extractor (*BIOMEDIX*®, Dietzenbach, Germany, Fig. 2) is positioned in the annulus (Fig. 1) to harvest the bone cylinder (Fig. 2). This results in a femoral bone tunnel with a diameter of 8.24 mm and a cancellous bone cylinder with a diameter of 7.16 mm × 25 mm (Fig. 2). Depending on the graft diameter, the femoral tunnel is asymmetrically dilated (Table S1, Fig. 3) and thus the graft bed is generated. The opposite cortex is drilled with a 2.3 mm drill wire (Smith + Nephew Inc. Andover, MA USA) and then with the 4.5 mm Endobutton drill (Smith + Nephew Inc. Andover, MA USA). A feed line is parked. The correct position of the tunnel is checked via arthroscope.

**Fig. 4:**
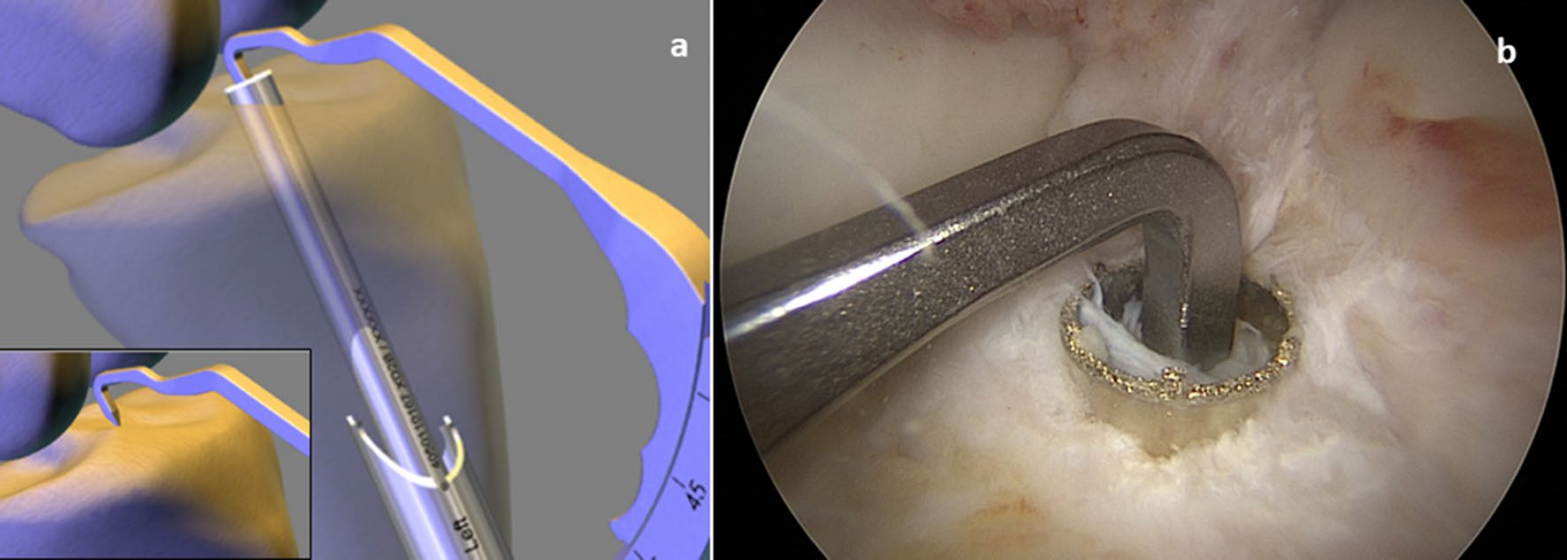
Tibial procedure: **a** tibial target device; Insert: onset on the ACL-stump; **b** hallow saw position after tibial procedure

**Fig. 5:**
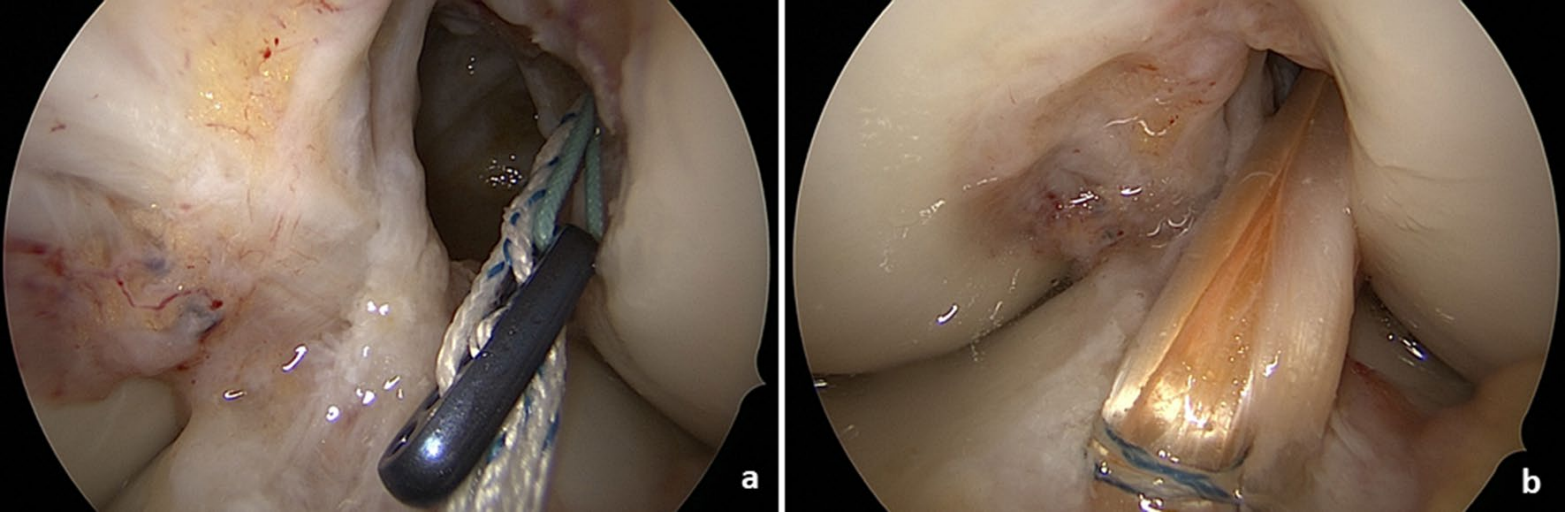
Graft placement: **a** dragging of the graft into the femoral bone tunnel by approximately 50%, view from top; **b** dragging into the tibial bone tunnel completed

Now the tibial guiding device is inserted (Fig. 4) and positioned so that the guiding hook is centered in the area of the tibial ACL stump (Fig. 4) and a bone tunnel with a diameter of 8.24 mm is created and a cancellous bone cylinder with a diameter of 7.16 mm × 30–40 mm is removed from it with the diamond *AlphaLock®-*Turbo-Cutter (*BIOMEDIX*®, Dietzenbach, Germany). The correct position of the tunnel is checked via arthroscope. Transtibial removal of the thread and attachment of the graft follows. The transplant is pulled through the tibial tunnel into the femoral tunnel, till the Ultrabutton (Figs. 5 and 7 clear arrow) passes the opposite cortex. Then the Ultrabutton (Smith + Nephew Inc. Andover, MA USA) is flipped and the graft is vigorously distally withdrawn. The Utrabutton threads are tightened and the transplant is pulled in completely to a depth of 25 mm by that procedure (Fig. 5).

**Fig. 6:**
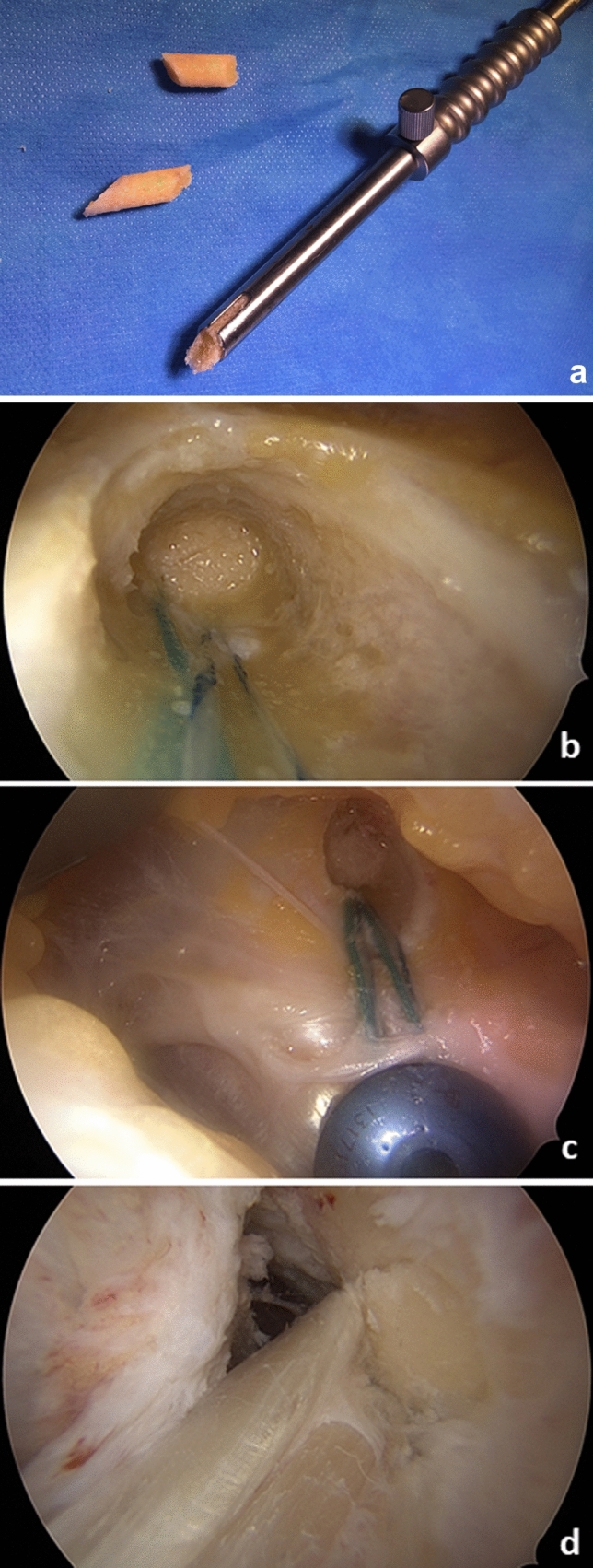
Bone blocks placement: **a** prepared bone blocks and bone block in bone block applicator; **b** first bone block in place (*tibial)*: **c** second *tibial* bone block in place. tibial hybrid fixation complete; **d** *femoral* bone block in place

**Fig. 7:**
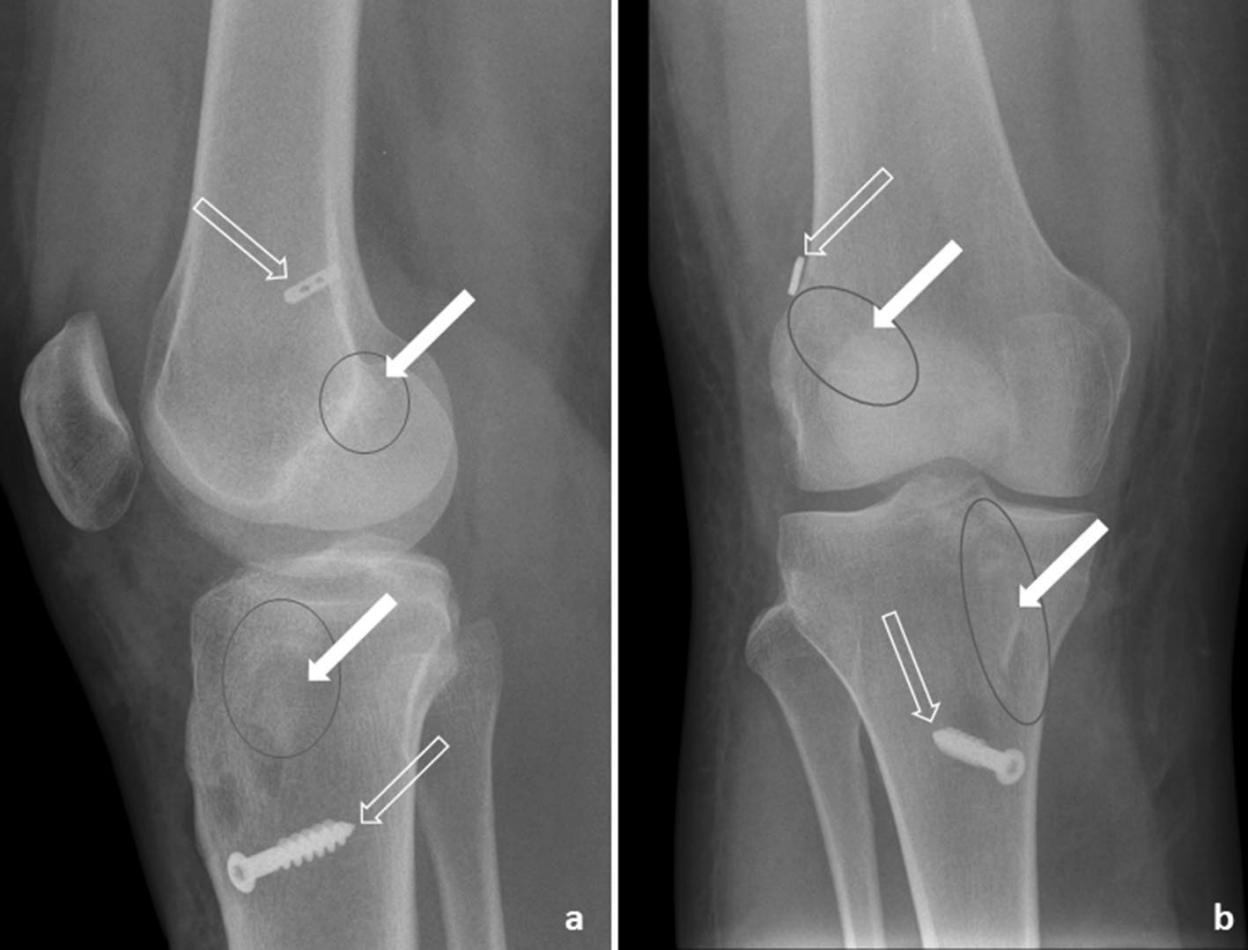
Xray 2 days after surgery (*Press-Fit-Hybrid®*-technique): **a** lateral view; **b** anterior/ posterior view; clear arrow: button and screw, white arrow bone block, for better visibility bone blocks are marked with black circles

The knee joint is flexed 30 times between 0 and 90° with maximum tension on the graft. To secure the graft, a hybrid extracortical fixation is performed. A 6.5 × 30 mm bollard screw (Nano Medical GmbH, Riedstadt, Germany, Figs. 6 and 7 lower clear arrow) is inserted after drilling approx. 15 mm distal to the tibial bone tunnel opening. In a 20° flexion-position, the distal pair of Hi-Fi threads (ConMed, Groß-Gerau, Germany) and the Ethibond-thread pair (Ethicon, Johnson & Johnson, New Brunswick, NJ, USA) are placed around the screw neck (Figs. 6 and 7 lower clear arrow) and firmly knotted together. The transplant is positioned by the use of a dilatator . Using the cancellous bone applicator (Fig. 6), the previously halved and prepared 7.16 × 40 mm cancellous bone cylinder (Fig. 6) is impacted in 2 steps in the tibial bone tunnel, which now fills and seals the tunnel nicely (Fig. 6). At the femur side, the transplant is positioned by the use of a dilatator too. The transplant now aligns anatomically to the dorso-caudal border of the lateral notch. Using the cancellous bone applicator (*BIOMEDIX*®, Dietzenbach, Germany, Fig. 6), a 7.16 × 17 mm cancellous bone cylinder is now decentered to the transplant and impacted into the femoral tunnel, so that in addition to the extracortical fixation, a press-fit fixation close to the joint is created. This does not require an intra-osseal implant and thus allows for the maximum contact area between graft and vital bone, producing maximum stability with minimal bone loss. The cancellous bone cylinder is hammered into the tunnel in such a way that the cortical part of the bone cylinder is at the same level as the cortical boundary of the femur/tibia (Fig. 6). Because the bone cylinders are pressed into the bone tunnel close to the joint, a bungee and windscreen wiper effect [17] is virtually eliminated, thus minimizing the risk of tunnel widening.

Intra-articular checks for correct positioning of the ACL transplant are conducted. The graft should be under adequate tensile stress and there should be no evidence of notch impingement at full extension. Finally, a partial synovectomy is carried out in all sections to reduce pain, and extensive irrigation is applied. A 10Ch Redon drainage (Fa. B. Braun AG, Melsungen, Germany) is inserted into the upper recess. 20 ml Naropin 0.75% is applied intraarticularly and subcutaneously. The skin is sutured with the Donati single button suture technique. Over the pes anserinus, a 12Ch Redon drainage (Fa. B. Braun AG, Melsungen, Germany) is inserted, with suturing of fascia, subcutis and dermis in intra-cutaneous technique. A final check for complete extension is performed and sterile, elasto-compressive wound dressings are applied.

### Rehabilitation scheme

The rehabilitation scheme differs between patients with only ACL reconstruction and patients with ACL reconstruction and meniscal refixation.

For ACL-only patients: From the second day until the end of the first week: isometric exercise, electrical muscle stimulation, active motion with CAMOped® (OPED, Valley, Germany) up to 0–0–90° 3 times a day for 10 min, VADOplex® (OPED, Valley, Germany) treatment 4 × a day for 30 min, ice several times per day, walking with crutches, weight bearing up to 30 kg, flexion of the knee as tolerated. From week 2: training in walking without crutches, full weight bearing as tolerated, muscle stimulation: 3 × 20 min per day, active motion to 0–0–110° with CAMOped® 3 × a day for 10 min. From week 3: full weight bearing, 3 × 20 min muscle stimulation per day, CAMOped® 4 × a day for 20 min. From week 5: muscle re-education training, physiotherapy, strength training, swimming, relaxed cycling on Ergometer. From week 7: endurance training, neuromuscular coordination training, cycling, inline-skating. From week 12: jogging/running, neuromuscular coordination training. Agility/ endurance/ strength training.

The rehabilitation scheme for patients having additional meniscal re-fixation was similar. The exceptions were: from the first week until week 4 only passive motion to 0–0–70°, weight bearing only 10–20 kg, walking with crutches, innervation education. From week 3: flexion to 0–0–90° as tolerated, weight bearing with half the body weight. After 4 weeks: full weight bearing as tolerated. From week 5: free movement, isometric exercise, no passive extension, active movement training with CAMOped® 4 × a day for 20 min, muscle stimulation 3 × a day for 20 min. From week 7: gradually walking without orthosis, no load in bending position up to the 12th week. Physiotherapy. For both groups: The “Return-to-Play-Test” was performed after 6 months. If successful a gradual start with professional sport was initiated. Competitive sport was permitted after either 9 months (ACL-only patients) or 10–12 months for ACL + meniscal refixation.

### Data acquired

Duration of surgery, complications, such as infections, fever, hematomas, follow-up, re-rupture rate, meniscal lesion after reconstruction, subjective assessment of patient outcome, return to work and to amateur sport were recorded. Knee stability was assessed with the Lachman-test, Pivot-shift-test and instrumental laxity measurements via Rolimeter® (Aircast Europa GmbH, Neubeuern, Germany) and range of motion (Neutral-0-Methode, https://www.dguv.de/medien/formtexte/aerzte/f_4224/f4224.docx were recorded in a questionnaire before surgery, 6 weeks, 3 and 6 months after surgery. These results were recorded long time before the data analysis was planned. Thus, even evaluated by the operating surgeon only, a bias can be excluded due to the fact that techniques were not used in parallel, except for a very short interim period, thus comparison of results was not possible at the time of interpretation.

Re-rupture, secondary meniscal injury, subjective patient assessment and return to work and sport were evaluated in the final follow-up (4 and 5 years for the PFH- and the IF-group, respectively) for all patients.

### Calculations and statistical analysis

The data analysis is only descriptive. The main focus of the retrospective data analysis is on re-rupture rate and secondary meniscal lesion. Due to the low number of re-ruptures and postsurgical meniscal lesion in the control group (below 10%), it is not expected to find significant differences. Log Rank test was used for analyzing the significance of the survival curve. The power for the re-rupture rate was calculated with 0.21. A power analysis showed, that over 500 patients in each group would be necessary to achieve significant differences for re-rupture rate and secondary meniscal lesion or, in the treatment group, there should be no incidence, which is fare from daily surgical practice. A power of 0.8 was assigned to be enough power to show significant differences The power for all significant values in this article was at least 0.86. Tendon size prediction was calculated according to Ramkumar et al. [38]. The relationship between re-ruptures and the ratio of folded diameter to patient height/weight/BMI was calculated according to Magnusson et al. [29].

Values are given as mean ± SD, with range, or median values, calculated with OriginPro, Version 2021 (OriginLab Corporation, Northampton, MA, USA). Because of non-Gaussian-distribution, Kruskal–Wallis-Tests were carried out. Where appropriate, contingency tables were used and significant differences calculated with Fisher’s-Exact-Test or Pearson-Chi-Square-Test and ODDS-ratios were calculated. Kaplan–Meier-survival-curves were used to depict revision free survival of the ACL-reconstruction and Hazard-function was used for the cumulative risk of re-rupture. A *p* value < 0.05 was defined as significant. When values were missing the number of observations were presented.

### Funding

The study was funded by the first author in total.

## Results

### Patient data (Table 1)

**Table 1:** Demographic and pre-operative data

Parameter	Interference screw technique	*Press Fit- Hybrid®* technique	*p* value
No of patients	100	100	
Age (years) *	36.17 ± 12.27	35.38 ± 12.10	0.53144
Range	18—57	18–66	
BMI (kg/m^2^**)** *	25.66 ± 3.43	25.10 ± 3.65	0.12673
Range	18.82–37.04	19.37–40.26	
Male	57	59	
Female (% female)	43 (43)	41 (41)	
Right knee/left knee	52/48	61/39	
Traumatic rupture (y/no/no data)	93/1/6	95/1/4	
Injured during:			
Sport	79	81	
Traffic	1	5	
Work	2	3	
Home	2	4	
Others	9	4	
No data	7	3	
Time to surgery*	24.97 ± 70.2 months	24.07 ± 69.4 months	0.65563
Range	23 days to 36 years	26 days to 35 years	
Primary surgery (*n*)	85	87	
Associated injuries			
Medial meniscal lesion (*n*)	48	34	
Lateral meniscal lesion (*n*)	42	27	
Cartilage lesion (*n*)	47	40	
Others (*n*)	2	0	
Association between time to surgery and meniscal lesion	Time to surgery ≤ 180 days > 180 days	Time to surgery ≤ 180 days > 180 days	
Medial meniscal lesion	30 18	24 10	
No medial meniscal lesion	44 8	44 22	
	*p* = 0.01342		
Lateral meniscal lesion	33 9	18 9	
No lateral meniscal lesion	41 17	49 24	

^*^Values are given as the mean and standard deviation

Patient groups were not significantly different with respect to age, BMI and gender, affected side, associated injuries, percent of traumatic rupture, the reason for the injury, the percentage of primary surgery, time to surgery and distribution between rehabilitation schemes (Table 1). Patients in the IF-group, who were operated more than 6 months after ACL rupture had a significant (*p* = 0.01342 higher rate of medial meniscal lesion at the time of surgery (Table 1).

### Postoperative outcome (Table 2)

**Table 2:** Post-Surgery Outcome

Parameter	Interference screw technique	*Press Fit- Hybrid®* technique	*p* value
Duration of surgery (minutes) *	46.93 ± 10.00	51.77 ± 7.60	< 0.0001
Range (minutes)	29–88	37–74	
beginning of new method (minutes)		56	
after 20 months (minutes)		48	
slope		− 0.01247	0.01861
Complications	2 -post-operative infection (Staphylococcus Epidermidis), arthroscopic irrigation -fever, no infect arthroscopic irrigation	1 -fever, no infect, arthroscopic irrigation	
Last follow up*	1917 days (5.3 years) ± 269 days	1519 days (4.2 years) ± 131 days	< 0.0001
Re-rupture: yes/no (%)	9/91 (9)	4/96 (4)	
Re-Trauma (yes/no)	7/2	3/1	
Meniscal injury after surgery	9	6	
Re-ruptures in patients with			
medial meniscal injury before surgery	4 (8.3%)	1 (2.8%)	
lateral meniscal injury before surgery	3 (7.1%)	0 (0%)	
General condition after revision surgery			
Better	1	2	
Same	11	11	
Worse	2	0	
Much worse	1	0	
Age (years) *			
No ruptures	36.59 ± 12.23, *n* = 91	35.99 ± 11.397 *n* = 96	
Ruptures	31.89 ± 12.50, *n* = 9	20.75 ± 1.26, *n* = 4	
No ruptures vs. ruptures		*p* = 0.00873	

^*^The values are given as the mean and standard deviation

The mean duration of surgery was 47 min and 52 min for the IF-group and PFH-group (*p* < 0.0001), respectively. Due to the introduction of the new surgical procedure (PFH), the duration of surgery was longer at the beginning (56 min) than after 20 months (48 min, *p* = 0.01861) and returned to the time of surgery in the IF-group (47 min).

**Fig. 8:**
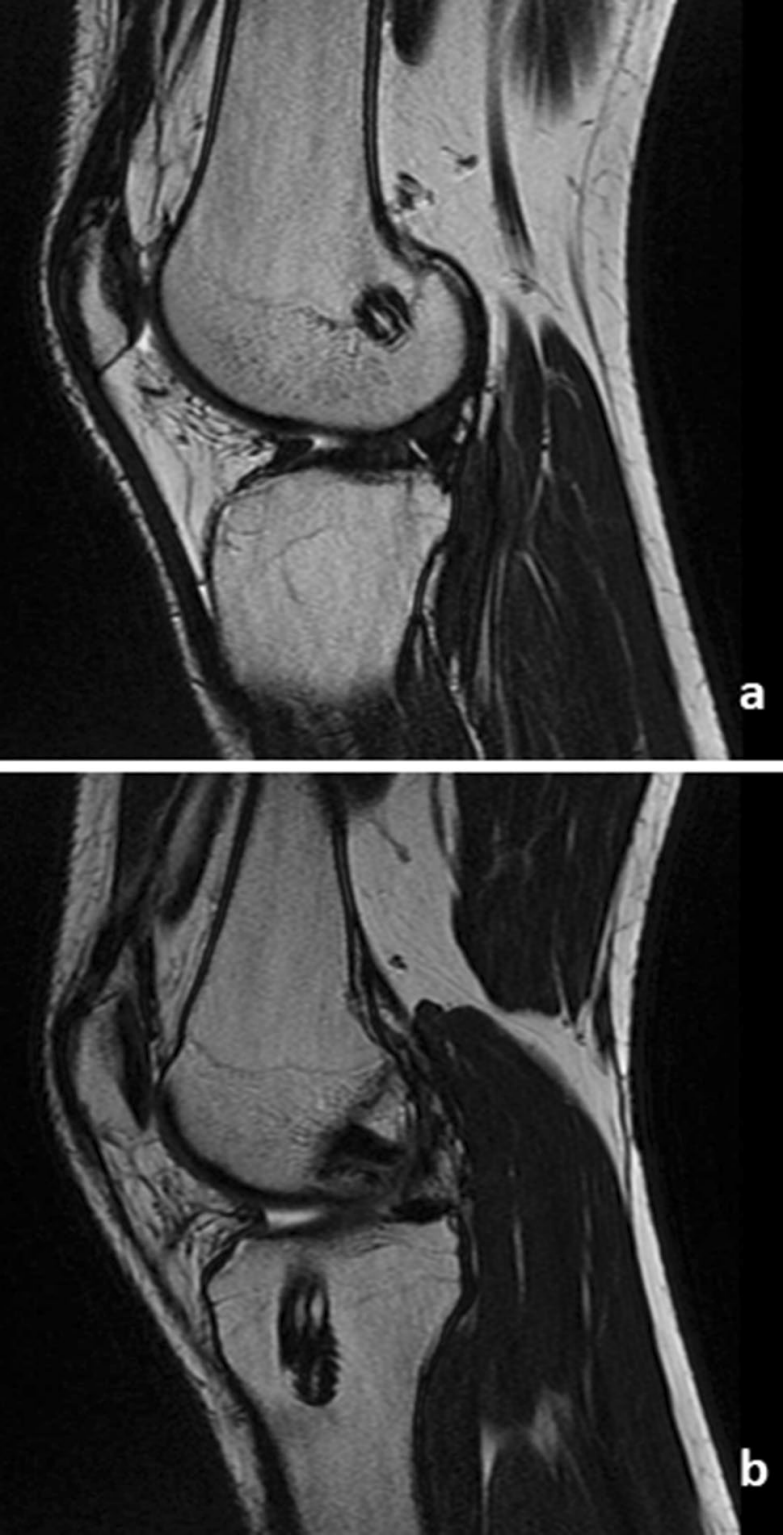
MRT Interference screw technique 2 years after surgery, lateral view: **a** femoral interference screw is still well visible; **b** tibial interference screw still well visible

**Fig. 9:**
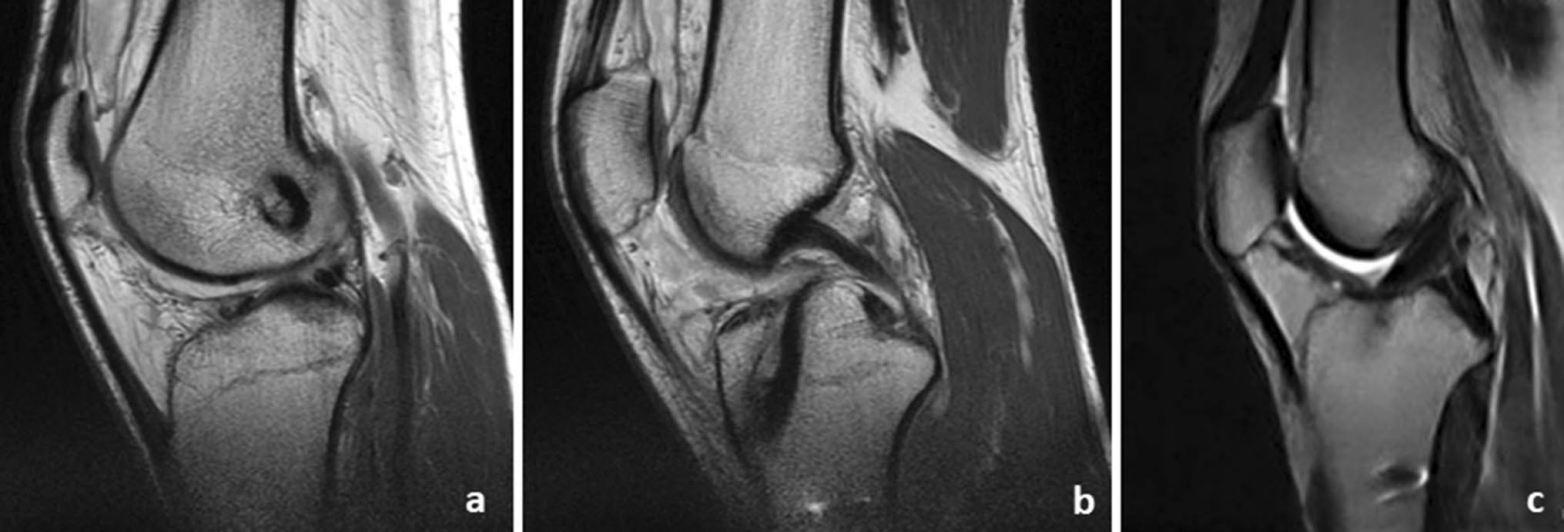
MRT *Press-Fit-Hybrid®*-technique 2 weeks after surgery, lateral view **a** femoral tunnel and bone cylinder; **b** tibial tunnel with bone cylinder and graft; **c** ACL graft

The interference screw is not converted to bone as depicted in Fig. 8, two years after surgery. The screw is still well visible in the femoral as well as in the tibial tunnel (Fig. 8). While 2 weeks after surgery using the *Press-Fit-Hybrid®*-technique the still vitally looking bone block started to integrate to the surrounding bone and the graft is well in place (Fig. 9).

There were 2 complications related to the knee surgery in the IF-group and 1 in the PFH-group (Table 2).

The mean follow-up duration was 5.3 years and 4.1 years for the IF and PFH-group, respectively, due to the subsequent use of the techniques (Table 2).

Re-ruptures were observed in 9 patients (9%) in the IF-group and in 4 patients (4%) in the PFH-group. Adequate re-trauma was recorded for 7 patients in the IF-group and for 3 patients in the PFH-group. Most re-ruptures occurred after primary surgery, with only 1 re-rupture after revision, in both groups. Gender distribution and BMI in patients with re-rupture did not differ to the total patient distribution.

**Fig. 10:**
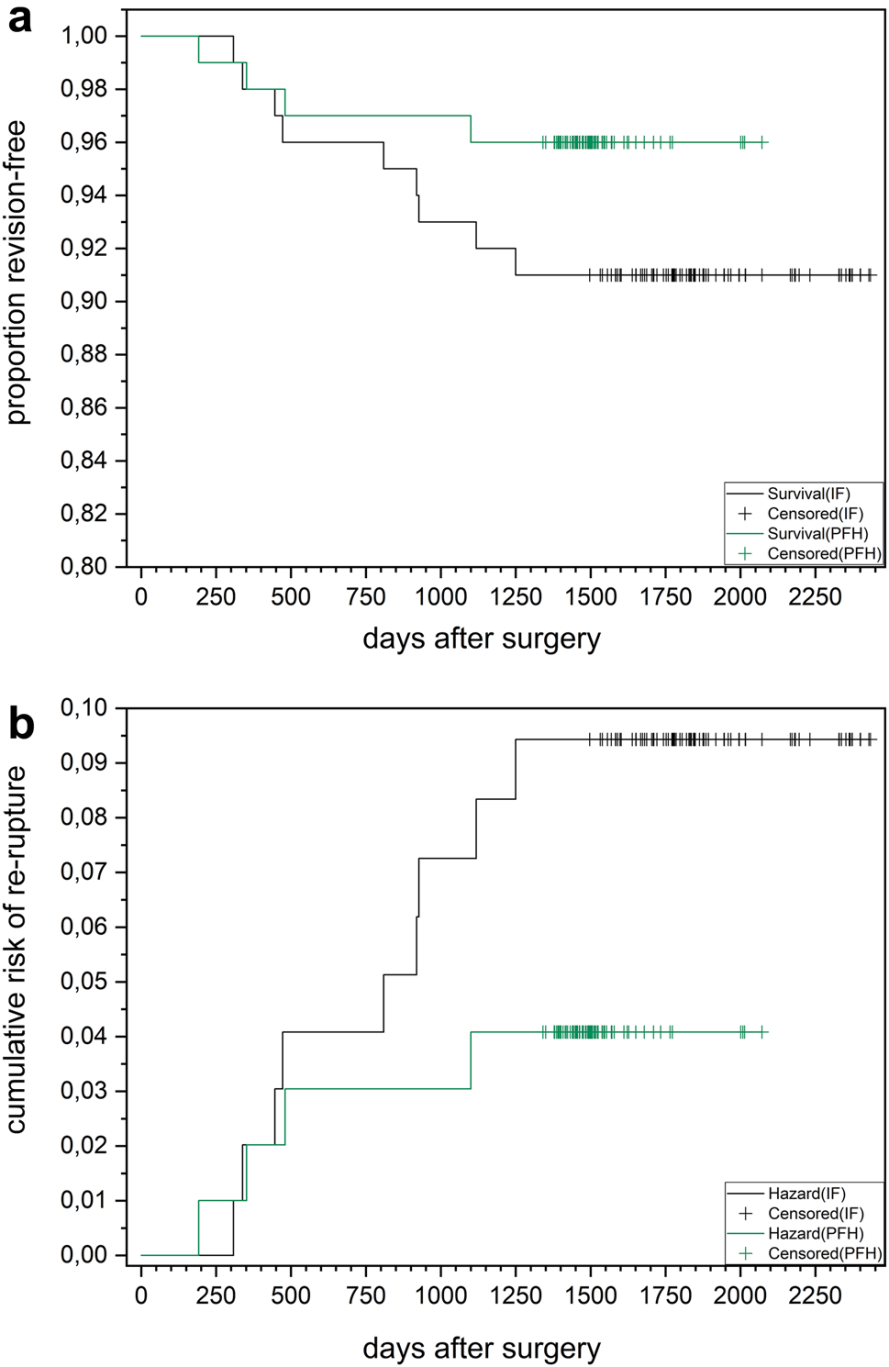
Kaplan Meier analysis: **a** Proportion revision free survival; **b** cumulative risk of re-rupture for the interference-screw group (IF, black) and the *Press-Fit-Hybrid®*-group (PFH, green), *p* = 0.15679

Kaplan–Meier survival analysis revealed, that the proportion of revision free survival (Fig. 10a) was higher in the PFH-group and the cumulative risk of re-rupture (Fig. 10b) was lower in the PFH-group during the time investigated, even not significant (*p* = 0.15679). For patients in the IF-group, the hazard ratio was 2.3 to have a re-rupture (not significant, *p* = 0.2507). Follow up time in the PFH-group (shorter follow-up) was 3 months longer than the latest re-rupture recorded in the IF-group. Data analysis was performed until end of 2019. No further re-rupture was reported to the surgeon in both groups until now (2021). Due to this observation, it is not assumed that re-ruptures occurred after a time point of 4 years, in both groups.

Time to re-rupture was similar for both groups (732 and 531 days for the IF- and PFH-group, respectively, *p* = 0.4404). The not significant lower time to re-rupture in the PFH-group is due to 1 re-rupture in a professional athlete after 192 days, who was not compliant in performing professional contact sport 6 months after surgery. Excluding this patient from the calculation would lead to a time to re-rupture of 644 days, which is very similar to that observed in the IF-group, but would lead to a bias for data interpretation.

Secondary meniscal injury post-surgery was 30% lower in the PFH-group with 6 and 9 in the IF-group. Re-rupture rate in patients with meniscal lesions in addition to ACL rupture, at the time of surgery, was similar compared to all treated patients.

**Fig. 11:**
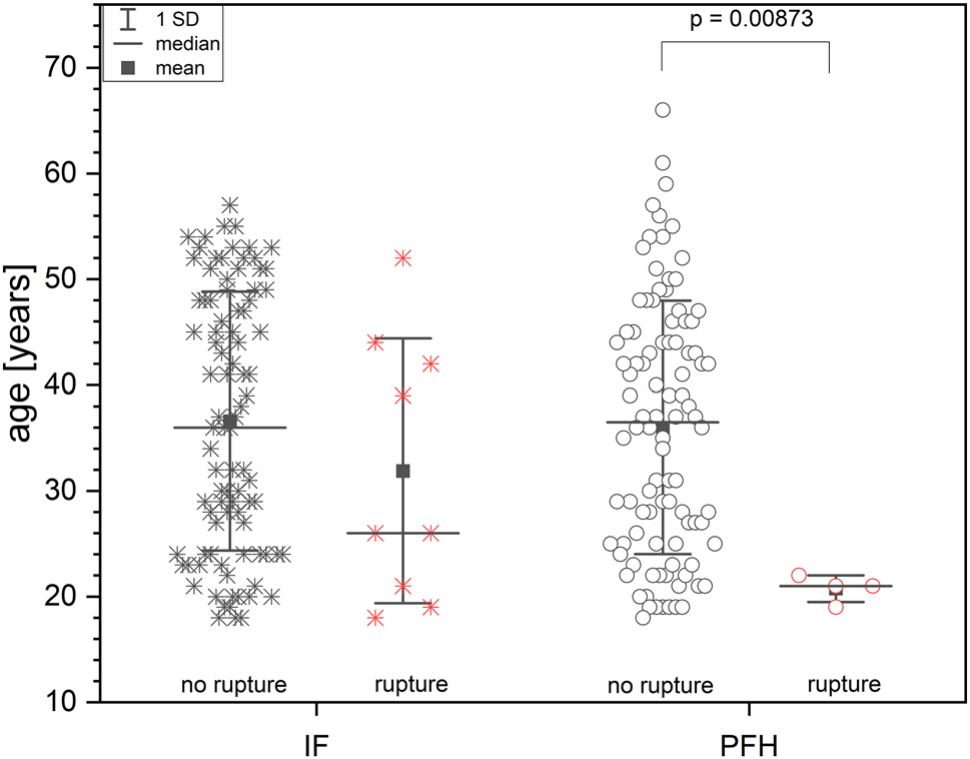
Patient age: Patients are divided in rupture/no rupture group, PFH: *Press-Fit-Hybrid®*-group; IF: Interference-screw-group) There is no difference in patient age between the IF and PFH in the no-rupture group (*p* = 0.59889), and between the 2 re-rupture groups (*p* = 0.18599) but there is significant difference between patients with rupture and patients without rupture in the *Press-Fit-Hybrid®* group (p = 0.00873), presented as mean ± SD and median

In the PFH-group, ruptures occurred exclusively in patients younger than 24 years, whereas in the IF-group, patients up to 52 years old had re-ruptures (Fig. 11). In the PFH-group there was a significant difference of age between the patients with and without re-ruptures (Fig. 11, Table 2, *p* = 0.00873).

### Post-operative knee stability: (Table 3)

**Table 3:** Post-surgery Knee Stability

Parameter	Interference Screw technique	*Press Fit- Hybrid®* technique	*p* value
Rolimeter difference*			
Pre-OP	4.82 ± 2.12, *n* = 97	5.42 ± 2.09, *n* = 99	0.0094
Range (mm)	0–14	0-11	
Last FU	0.12 ± 0.39, *n* = 85	0.13 ± 0.40, *n* = 92	
	180 days after surgery	176 days after surgery	
	*p* < 0.0001	*p* < 0.0001	
Range (mm)	0–2	0–2	
	6 patients 2 mm	8 patients 1 mm	
	2 patients 2 mm	2 patients 2 mm	
Lachman test 0 1 2 3	Pre last FU 0 96 8 0 89 0 2 0 180 days	Pre last FU 0 100 3 0 96 0 1 0 173 days	
Pivot shift test 0 1 2 3	Pre last FU 0 94 4 0 72 0 2 0 157 days	Pre last FU 0 100 6 0 81 0 0 0 174 days	
Degrees of flexion*(neutral-0-method)			
53/ 56 days after surgery	118.60° ± 15.49°, *n* = 100	120.80° ± 16.91°, *n* = 100	
118/108 days after surgery	130.35° ± 9.22°, *n* = 85	133.07° ± 8.72°, *n* = 91	
235/188 days after surgery	132.30° ± 8.77°, *n* = 52	136.09° ± 5.35°, *n* = 69	0.00529
Extension deficit at last follow up	10° in 2 patients 5° in 6 patients	5° in 6 patients	

^*^The values are given as the mean and standard deviation; Pre: Pre-surgery; FU: follow up

Pre-surgery the Rolimeter® difference was significantly lower (*p* = 0.0094) in the IF-Group (4.8 mm ± 2.1 mm) than in the PFH-Group (5.4 mm ± 2.1 mm). At final follow-up, the Rolimeter® difference was near to zero in both groups. Significant lower Rolimeter values were obtained at last follow up in both groups (*p* < 0.0001) with no difference between the groups post-surgery (Table 3). There were 8 patients in the IF group and 10 patients in the PFH-group with Rolimeter differences between 1 and 2 mm, which did not lead to meniscus lesion later-on.

Pre-surgery, Lachman and Pivot-shift-test values were positive in all cases. At final follow-up data were available from 96 patients in the IF-group and from all in the PFH-group. Patients in both groups had negative Lachman and Pivot-shift-test values. No difference was observed between the surgery groups (Table 3).

The PFH-group showed a higher degree of flexion (133.1° ± 8.7° vs. IF-group:130.4° ± 9.2°) already 3 months after surgery, which further increased after 6 months (132.3° ± 8.8° and 136.1° ± 5.4° for the IF and PFH-group, respectively, *p* = 0.00529). An extension deficit of 10° was recorded in 2 patients in the IF-group, an extension deficit of 5° was measured in 6 patients in both groups.

### Return to work and amateur sport

Return to the previous work model (full-time/part-time) was observed for 96% in the IF-group and 100% in the PFH-group. Return to the level of amateur sport after surgery was similar in both groups, with a level of pre-surgical activity of 81% and 81.5% for the IF- and PFH-group, respectively.

### Tendon size prediction and relation to rupture

Tendon size prediction according to Ramkumar et al. [38] demonstrated, that when the measured diameter during surgery was larger than the calculated diameter, there were no re-ruptures in the PFH-group and only 1 re-rupture in the IF-group. Ruptured and non-ruptured graft size was similar in both groups.

We did not observe any correlation between re-ruptures and the ratio of folded diameter to patient height/weight/BMI [29].

## Discussion

The most important observation of this retrospective analysis is that the *Press-Fit-Hybrid®-*technique, using hamstring tendon, shows a trend to lower re-rupture rates (44% of the re-ruptures in the interference screw group) and also a trend to low secondary meniscal lesions after surgery (67% in comparison to the interference screw-technique).

The optimal technique for ACL reconstruction, especially using Hamstring grafts, is still debated [17]. The use of press-fit technique for both, femoral and tibial fixation is less described [14]. The use of hamstring tendon is known to be associated with lower donor site morbidity but higher re-rupture rate [3]. The lower rate of donor side morbidity was the reason for using hamstring grafts, when available. The re-rupture rate of nearly 10% was the reason for trying another method for fixation. The herein described *Press-Fit-Hybrid®-*technique uses on both sides, femoral and tibial, autologous bone plugs for fixation of the hamstring tendon. This technique makes revision easier since drill tunnels are filled with bony material and one-side revision is possible without the need of spongioplasty [7]. This observation is important to surgeons performing ACL-reconstruction, especially to those treating young patients or performing revisions. The *Press-Fit-Hybrid®-*technique presented, works with the tools provided for standardized bone tunnel dilatation and in accordance with the supplied matrix, associating tunnel and graft sizes. Such a standardized technique leads to an optimal impaction of the bone bed and a consistent level of fixation between the bone cylinder, the bone bed and the graft, thus maximizing the contact zone. The double security system (autologous bone cylinder and hybrid fixation) is probably responsible for the presented results. Introducing the new method increased the time of surgery, but after 20 months, duration of surgery returned to values of the old method. Others describe an operation time of 71–76 min [21] or more than 90 min [43].

Previous studies report a re-rupture rate for the interference-screw-technique of 9–11%, [43] 14.5%, [39] 14.7% [20], 16% [35], and for press-fit-technique with quadriceps-tendon-patellar-bone of 9% [6], which is above the percentage observed in the presented study, with 4% and 9% for the PFH-group and IF-group, respectively. Kampinski et al. [27] did not observe any rerupture in a small group of patients, when using the interference screw technique with hamstring tendon or the press fit technique on one side with quadriceps tendon, but the follow up time was only 2 years. Due to the low number of re-ruptures in the control (IF)-group, even with a decrease of re-ruptures to 44% in the PFH-group, significant differences could not be shown. From the 4 patients suffering a re-rupture in the PFH-group, 1 had a re-rupture 6 months after surgery, whilst participating in professional contact sport. The time to return to professional sport may be a key parameter. Others report a gradual return to sports activity after eight to 12 postoperative months and after one year without any restrictions [20]. The return to sport percentage was 81% in both groups. Other report 91% [33]. The difference may be due to the percentage of athletes in the population studied. In our data set, female gender was not a predictor for re-rupture as described elsewhere [25].

The aim of the reconstruction is to regain ACL-stability and to prevent later meniscal lesion and arthrosis. Aiming for a reduction in meniscal lesion post-surgery is described to be related to the method used [43] and is a major reason to change the surgical procedure. In our study, pre-existing medial and lateral meniscal lesions and cartilage lesions were similarly distributed between the 2 groups—and as reported previously [19, 27, 28, 43], therefore did not contribute to differences in the final outcome [20]. But as described by Ahlen et al. [2] there is a significant higher number of medial meniscal lesions in the IF-group, when patients were operated more than 180 days after injury. We observed 33% less secondary meniscal lesion in the PFH-group. Secondary meniscal lesion in the IF-group (9%) was as previously reported with 7.1% [43], 8%, [40], 6–10%, [19, 28] 12.5% [32] and 14% [12]. Similar results were reported when quadriceps tendon was used (4.3% [43]). In the IF-group 6 of the 9 secondary meniscal lesions were related to re-rupture. Three of these patients did not have meniscal lesions before the index surgery. Of the 6 patients in the PFH-group with a meniscal injury after the reconstruction surgery, a re-rupture was the cause of this injury in 2 patients and 2 had meniscal injury before the index surgery. Meniscal lesions were always addressed when present, this may explain the results observed in this study in respect to pivot shift data. Jacquet et al. [23] described non-repair of preoperative meniscal tear as high predictors for high grade pivot shift at follow up. We did not observe a correlation between pre-surgical meniscal injury and re-rupture as confirmed by others [26, 37, 39].

After surgery, laxity was significantly reduced in both groups (*p* < 0.0001). There was no difference between the groups at the last follow up. This is in keeping with previous reports [4, 22, 25, 27, 41, 43]. Eight patients in the IF-group and 10 patients in the PFH group had a Rolimeter differences of 1 or 2 mm, which signifies very light instability, but this is classified as negative Lachman and is as reported by others [7, 21, 27, 43]. Patients with 1 or 2 mm Rolimeter difference were not the patients experiencing secondary meniscus lesion during follow up interval.

Post-surgery Lachman- and Pivot-shift test were negative in all patients, where data were available and no difference could be observed between the groups. Previous studies have described a positive Lachman for 19% [31], and 36% of the patients [8]. No positive pivot shift [27] or a positive pivot-shift after surgery in 8–12%, [22], 15% [31] 18% [1], 21% [8] and 29–30% [43] of the patients are described too. The Rolimeter test is a more objective test than Lachman and Pivot shift, even it is done by the surgeon itself, Rolimeter difference is measured with an instrument. The negative Lachman and pivot shift is in line with the Rolimeter results obtained.

The degree of flexion 6 months after surgery was 132° and 136° for the IF- and PFH-group, respectively. This confirms the findings of Yari et al. [47] The PFH-group showed significantly (*p* = 0.00529) better results at more than 100 days post-surgery, providing patients with increased mobility. This might be attributed to faster healing in the PFH-group, as a result of a primarily biological reconstruction of the ACL without metal or resorbable screws.

Patient satisfaction, even subjective, is important to judge the outcome of a surgical method. In the PFH-group, all (100%) revision patients were either very satisfied or satisfied with the stability, whereas only 81% in the revision-IF-group reported being at least satisfied. Sarzaem et al. [41] describes 85% satisfaction in the press fit group. Even not statistically significant, the biological fixation system seems to improve patient satisfaction and supports the subjective impression of the orthopedic surgeon.

The mean follow-up duration was 4 years in the PFH-group and 5 years in the IF-group, which covers the time of risk of re-rupture and was also the basis for other studies [23, 43]. Other studies have concluded after 1 year [3, 4, 41] or after 2 years [13, 24, 43] of follow-up or even after shorter follow up time (6 months, [45]).

We could confirm results of previous publications [20, 35] demonstrating that young age is a risk factor for re-ruptures. In our study 11–13% re-ruptures occurred in patients below 24 years, whereas Webster et al. [46] described a 35% re-rupture rate in young (< 20 years) patients. We hypothesize, that young patients perform more sport with high risk for ACL-re-rupture, may not train their muscles sufficiently before returning to contact sport and are less compliant.

A high degree of return to the previous work model as recorded in our study, was also reported by Schindler et al. [42]. Longer sick leave was observed in patients with heavy physical work-load, an apparent correlation of longer sick leave with higher age was not significant.

Return to the level of amateur sport prior to surgery was over 70% in both groups and is consistent with values reported in previous publications (65% [5] and 73% [20]) for non-athletic patient groups.

We observed, that when the measured diameter of the graft is similar or higher than the calculated diameter, using the formula of Ramkumar et al. [38] there is no re-rupture in the PFH-group and only 1 re-rupture in the IF-group. This observation supports the hypothesis, that the graft diameter itself does not influence re-rupture rates, but the size of the graft in relation to the patient (height and BMI) does. Bedi et al. showed that increasing graft size did not increase knee stability at the time of reconstruction and did not compensate for poor knee stability arising from incorrect tunnel position [10]. Inadequate graft fixation is a reason for high re-rupture rates [1]. Akoto et al. [3] confirmed that graft fixation-technique influences results.

Using hamstring tendons for long time, with around 150 ACL reconstructions per year in the clinic of the first author, using the *Press-Fit-Hybrid®-*technique reduces the number of necessary ACL-revisions and reduces the surgery costs by 80.000 €/year for the insurance company, excluding costs for rehabilitation and sick leave, only in this one clinic.

In conclusion, performing ACL-reconstruction with the highly biological *Press-Fit-Hybrid®-*technique results in low re-rupture rates and low post-surgery meniscal injury, even in young patients and in revision surgery, whilst maintaining operation time and knee stability associated with interference screw methods. It seems likely that re-rupture rates could be further improved, if patient compliance could be increased. Especially in young patients (only these patients had re-ruptures in the *Press*-*Fit*-*Hybrid*®-technique) the awareness of the re-rupture risk associated with a hasty return to pre-injury sport levels could be a major determinant of outcome.

## Limitations

The retrospective evaluation of the data is a limitation. Another limitation is the chronological separation of the two treatment groups comprising patients treated either before, or after the change of the standard surgical procedure was made. Due to the low number of re-ruptures already in the control-group, the power of this data was not high enough to reach statistical significance, in respect to re-rupture rates.

## Supplementary Information

Below is the link to the electronic supplementary material.

Supplementary file1 (DOCX 724 KB)

Supplementary file2 (DOCX 16 KB)
