# Supplementary material for: Re-rupture rate and the post-surgical meniscal injury after anterior cruciate ligament reconstruction with the Press-Fit-Hybrid®-technique in comparison to the interference screw technique: a retrospective analysis of 200 patients with at least 3 years follow-up
Source: Arch Orthop Trauma Surg. 2022 Mar 15;143(2):935–49. doi: 10.1007/s00402-022-04368-7 (PMC9925573; doi:10.1007/s00402-022-04368-7)
Supplement: Supplementary file 2 — Supplementary file2 (DOCX 16 KB) [file 402_2022_4368_MOESM2_ESM.docx]

| Table S2 Rehabilitation scheme | | | | | | | | | |
| --- | --- | --- | --- | --- | --- | --- | --- | --- | --- |
|  | Partial weight bearing | Flexion/  extension | CAMO-ped® | CAMO-ped®  4x a day  20 min | Knee-orthosis | Muscle stimulation  3x a day for 20 min | Cycling/  Swimming/  jogging | Strength training | Coordination/  proprioception |
| ACL only | | |  |  |  |  |  |  |  |
| Week 1-2 | Half body weight | free | 3x 20 min |  |  | x |  |  |  |
| Week 3-4 | full | free |  | 4x 20 min |  | x |  |  |  |
| Week 5-6 | full | free |  |  |  | x | x/x/- | x |  |
| Week 7-8 | full | free |  |  |  | x | x/x/- | x | x |
| Week 9-12 | full | free |  |  |  | x | x/x/- | x | x |
| Week  13-16 | full | free |  |  |  | x | x/x/x | x | x |
| ACL and Meniscus refixation | | | | |  |  |  |  |  |
| Week 1-2 | 10/20 kg | 0-0-70° |  |  | x | x |  |  |  |
| Week 3-4 | Half body weight | 0-0-90° | 3x 20 min |  | x | x |  |  |  |
| Week 5-6 | full | free |  | 4x 20 min | x | x |  |  |  |
| Week 7-8 | full | free |  |  | (x) | x | x/-/- |  |  |
| Week 9-12 | full | free |  |  |  | x | x/x/- | x | x |
| Week  13-16 | full | free |  |  |  |  | x/x/- | x | x |
