# Supplementary material for: Re-rupture rate and the post-surgical meniscal injury after anterior cruciate ligament reconstruction with the Press-Fit-Hybrid®-technique in comparison to the interference screw technique: a retrospective analysis of 200 patients with at least 3 years follow-up
Source: Arch Orthop Trauma Surg. 2022 Mar 15;143(2):935–49. doi: 10.1007/s00402-022-04368-7 (PMC9925573; doi:10.1007/s00402-022-04368-7)
Supplement: Supplementary file 2 [file 402_2022_4368_MOESM1_ESM.docx]

Table S1 Standardized Dilatation Scheme

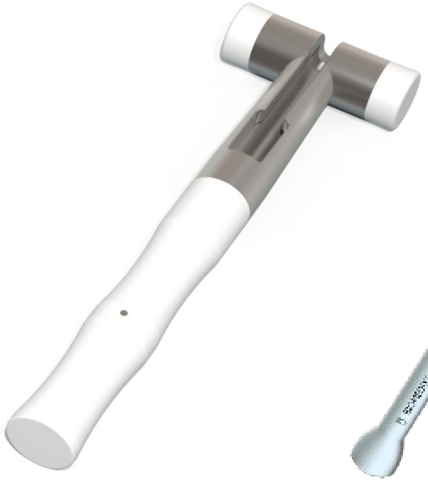

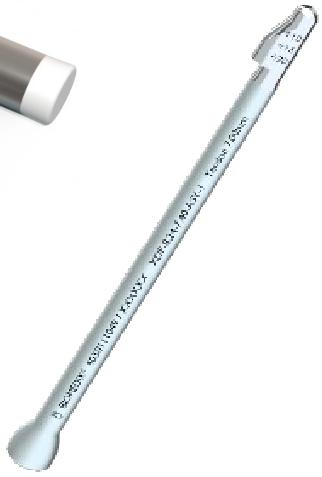

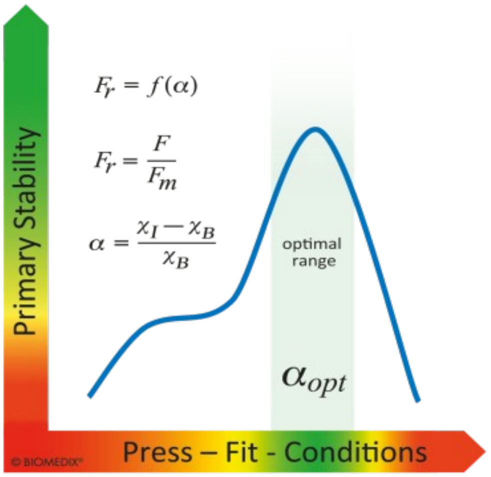

Press-Fit-Hybrid® **-** Femoral Dilatation

BIOMEDIX®
Advanced Biomedical Solutions

Folded Diameter

7,5 mm	**ASY_1**										
8,0 mm	ASY_1	**ASY_2**									
8,5 mm	ASY_1	ASY_2	**ASY_3**								
9,0 mm				SYM_A	SYM_B	SYM_C	**ASY_4**				
9,5 mm				SYM_A	SYM_B	SYM_C	ASY_4	**ASY_5**			
10,0 mm				SYM_A	SYM_B	SYM_C	ASY_4	ASY_5	**ASY_6**		
10,5 mm				SYM_A	SYM_B	SYM_C	ASY_4	ASY_5	ASY_6	**ASY_7**	
11,0 mm				SYM_A	SYM_B	SYM_C	ASY_4	ASY_5	ASY_6	ASY_7	**ASY_8**

Press-Fit-Hybrid® **-** Tibial Dilatation

BIOMEDIX®
Advanced Biomedical Solutions

Folded Diameter

7,5 mm											
8,0 mm											
8,5 mm	**ASY_1**										
9,0 mm				SYM_A	SYM_B	SYM_C	**ASY_4**				
9,5 mm				SYM_A	SYM_B	SYM_C	ASY_4	**ASY_5**			
10,0 mm				SYM_A	SYM_B	SYM_C	ASY_4	ASY_5	**ASY_6**		
10,5 mm				SYM_A	SYM_B	SYM_C	ASY_4	ASY_5	ASY_6	**ASY_7**	
11,0 mm				SYM_A	SYM_B	SYM_C	ASY_4	ASY_5	ASY_6	ASY_7	**ASY_8**

ASY: asymmetric dilatation; 1-8 instrument size, SYM: symmetric dilatation; A-C: instrument size
